# Sciatica Cured by Extract of Belladonna

**Published:** 1842-11

**Authors:** 


*Sciatica cured by Extract of Belladonna.*—The following interesting case is related in the *Bulletin Thérapeutique.*

A lieutenant in the French navy had long laboured under a very severe form of sciatica; the pain extended from the sciatic notch to the terminal branches of the nerve in the foot, and was of the most violent kind. Several remedies had been tried without effect, when M. Hiriart resolved on employing the extract of belladonna. The bowels were first cleared out by an active purgative; and the whole limb was then rubbed, several times during the day, with an ointment composed of one part of the extract to two of lard. After the fourth friction the patient experienced a creeping sensation in the limb, and some slight symptoms of narcotism appeared; he enjoyed, however, some sleep during the night. On the following morning the pain had shifted to the opposite limb, whence it was driven by the same means. The state of the bowels and stomach was regulated by gentle purgatives and proper diet, and in a short time the patient was completely cured of a disease from which he had previously suffered the most cruel torments.—*Prov. Med. and Surg. Journ.*, March 26, 1842.

